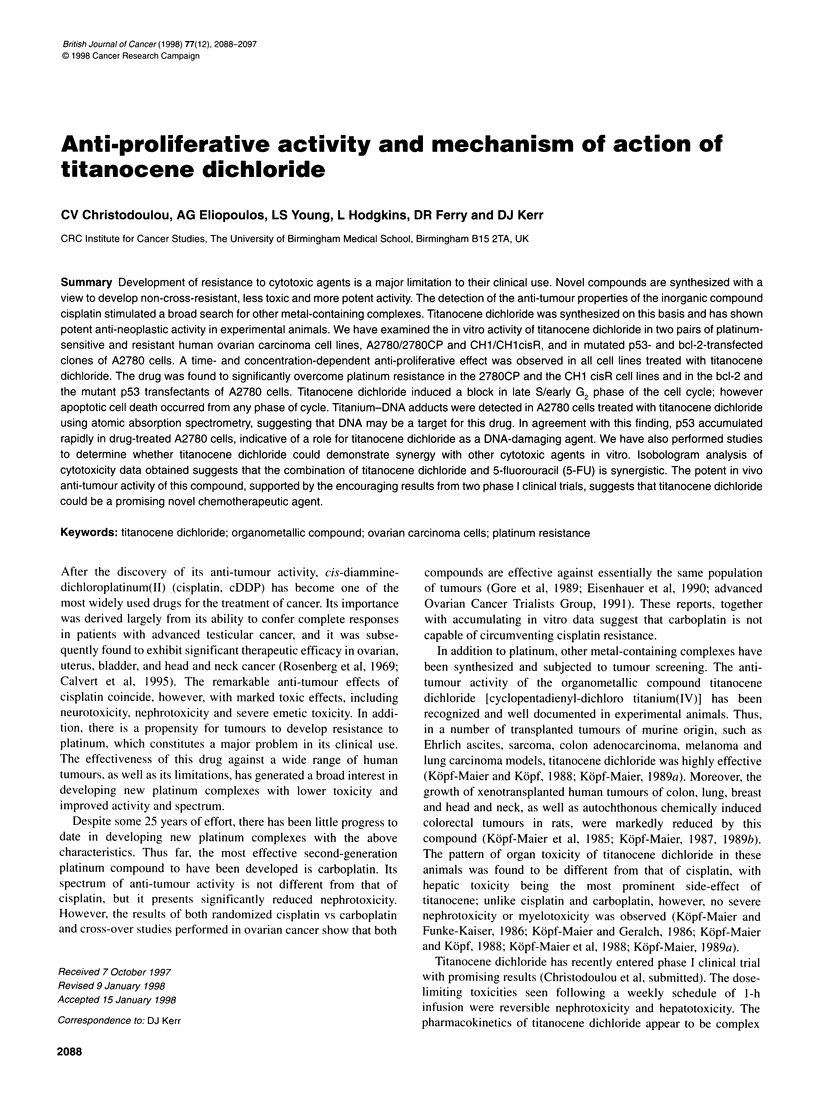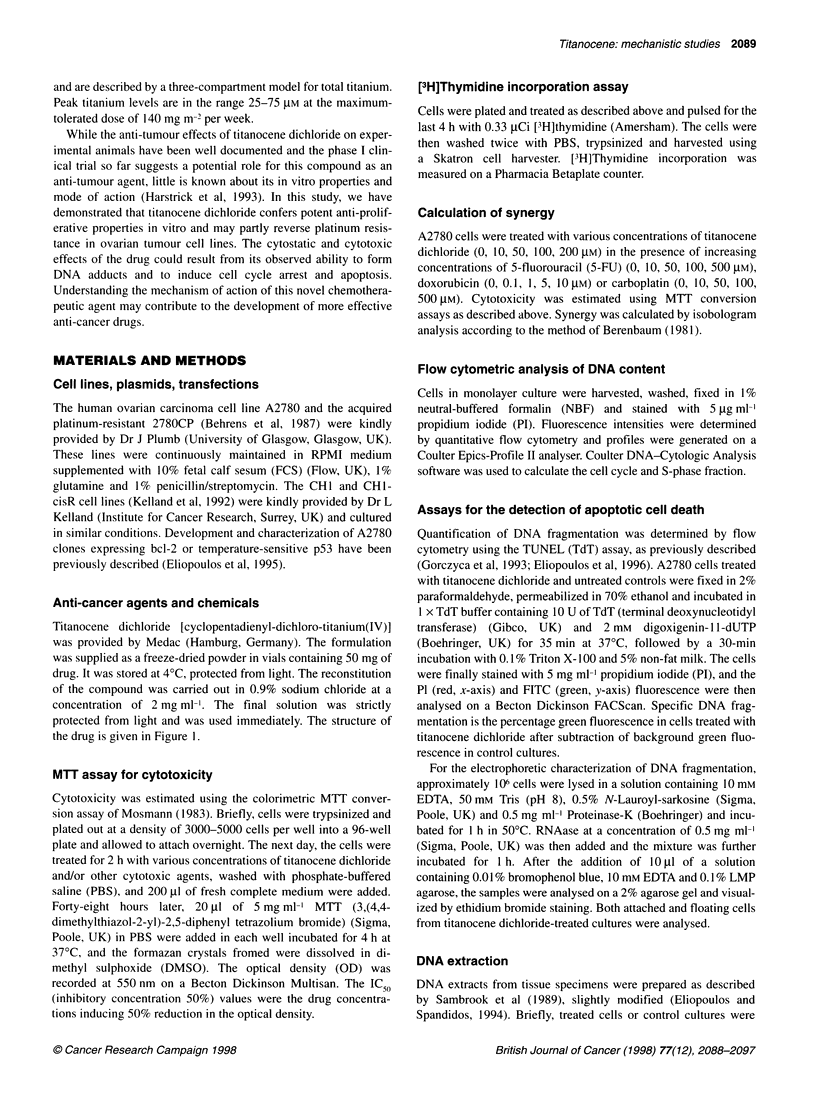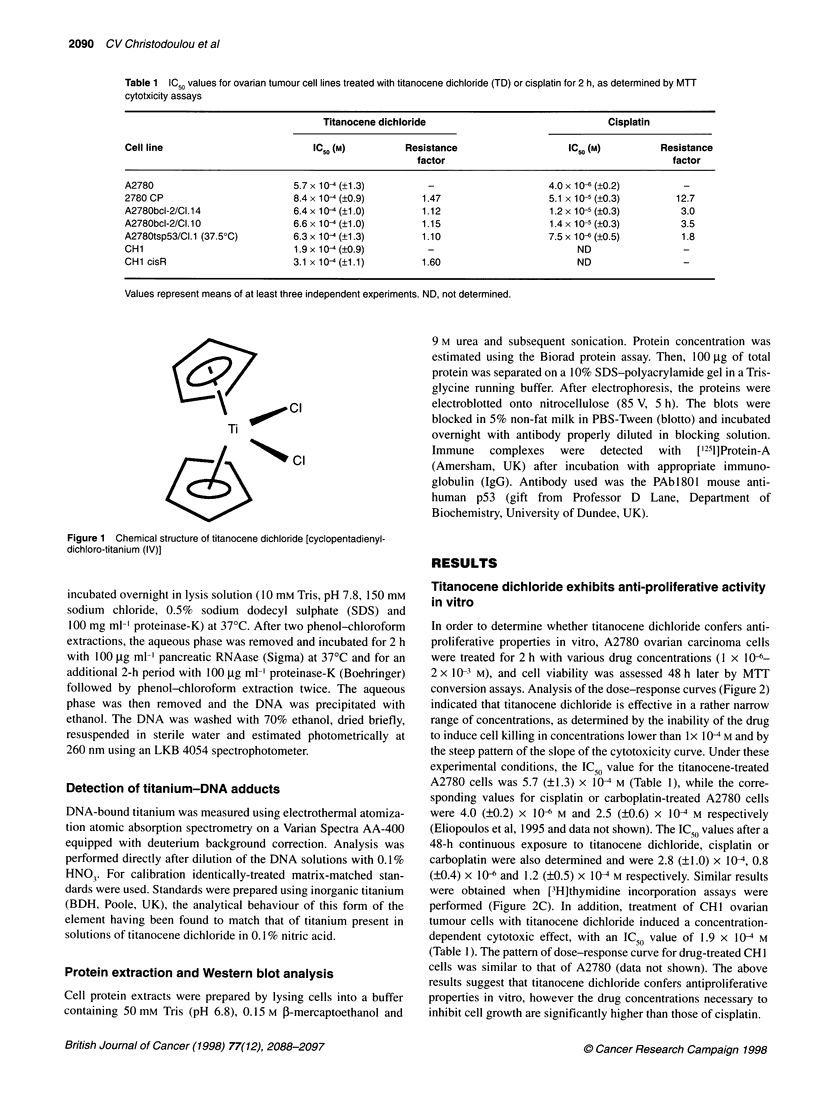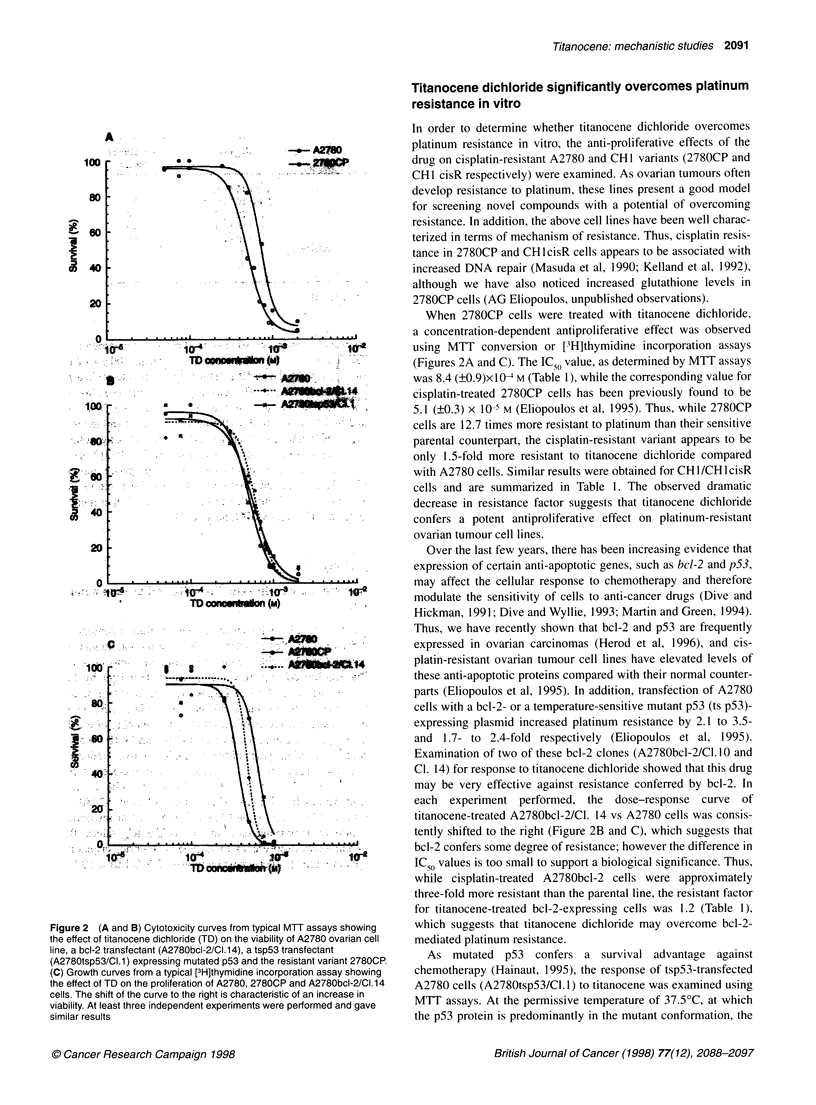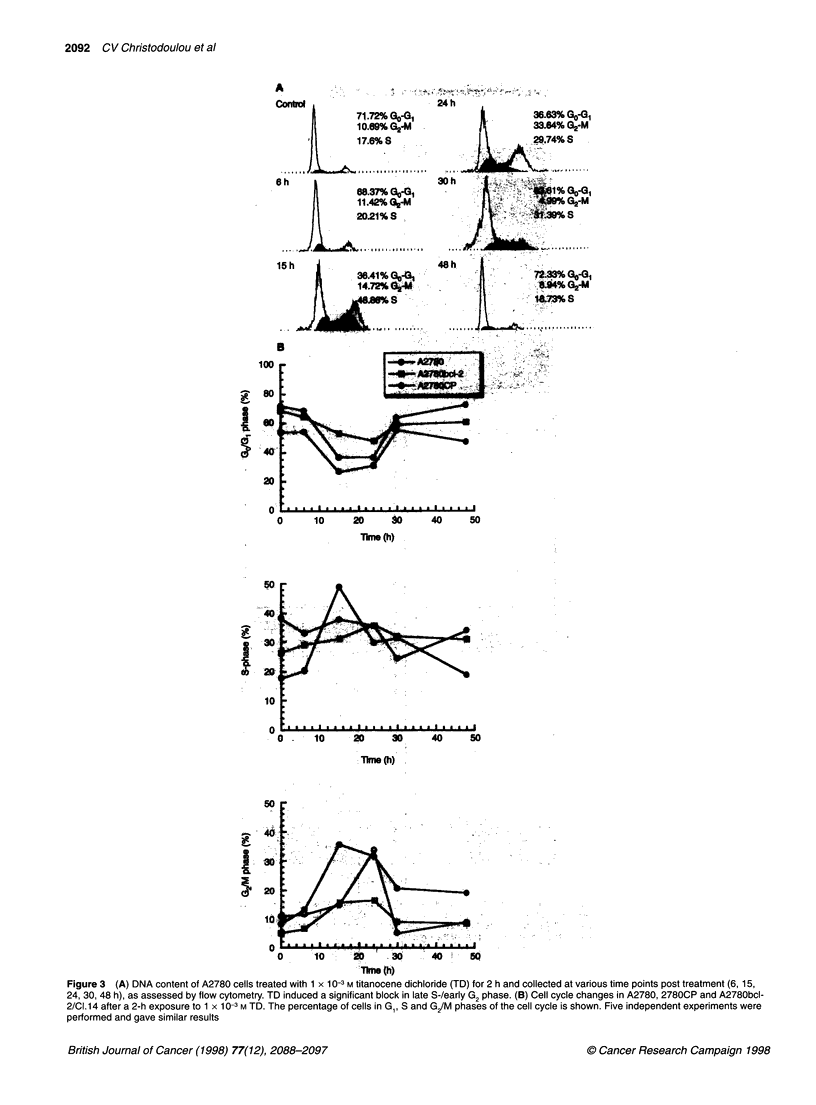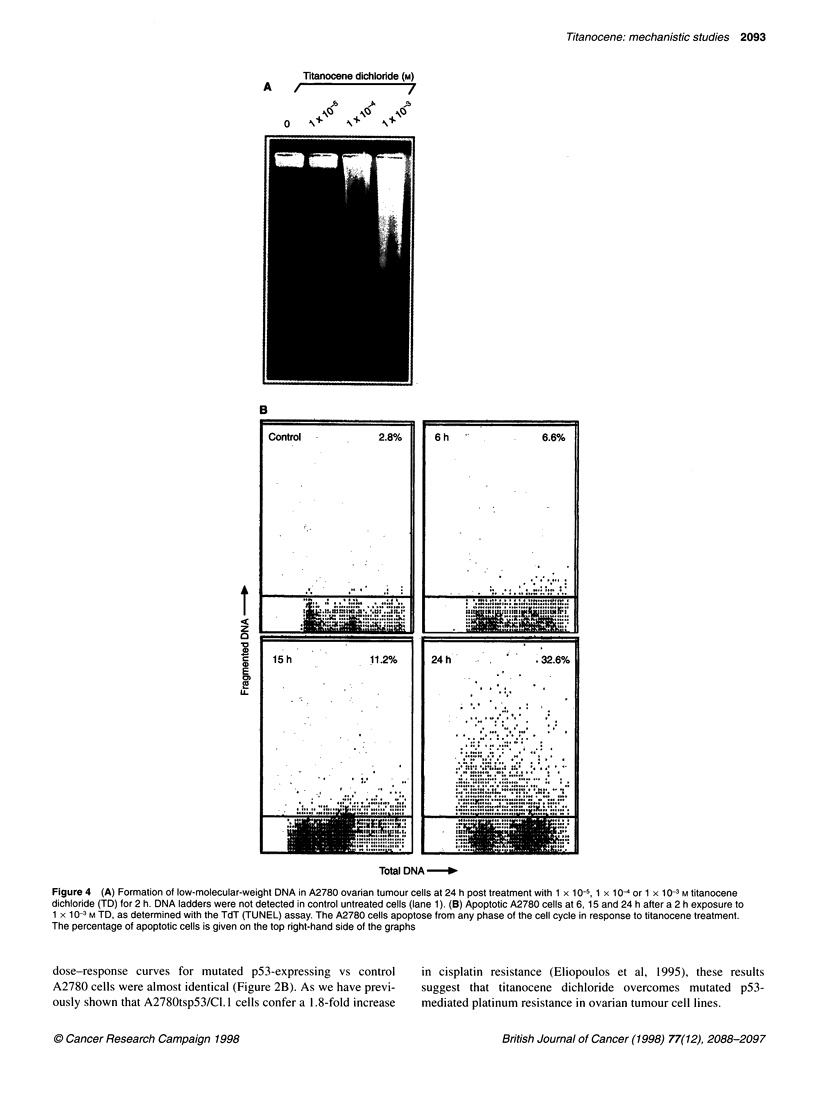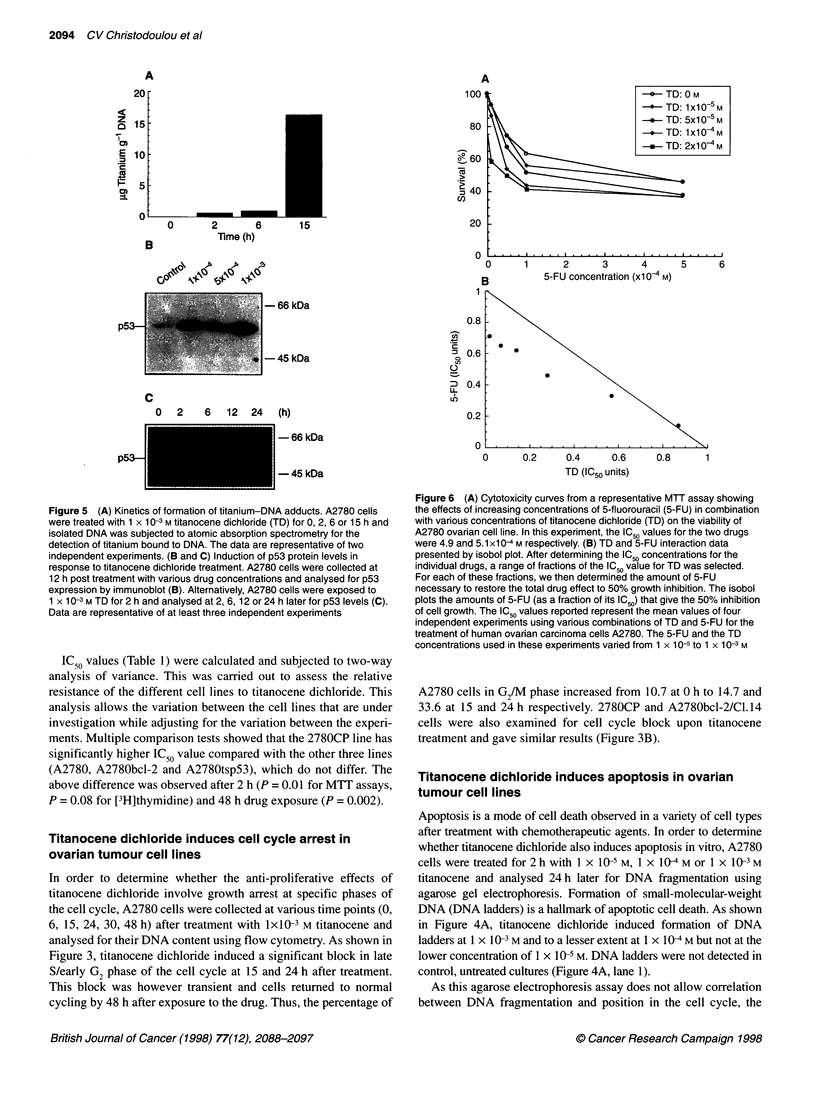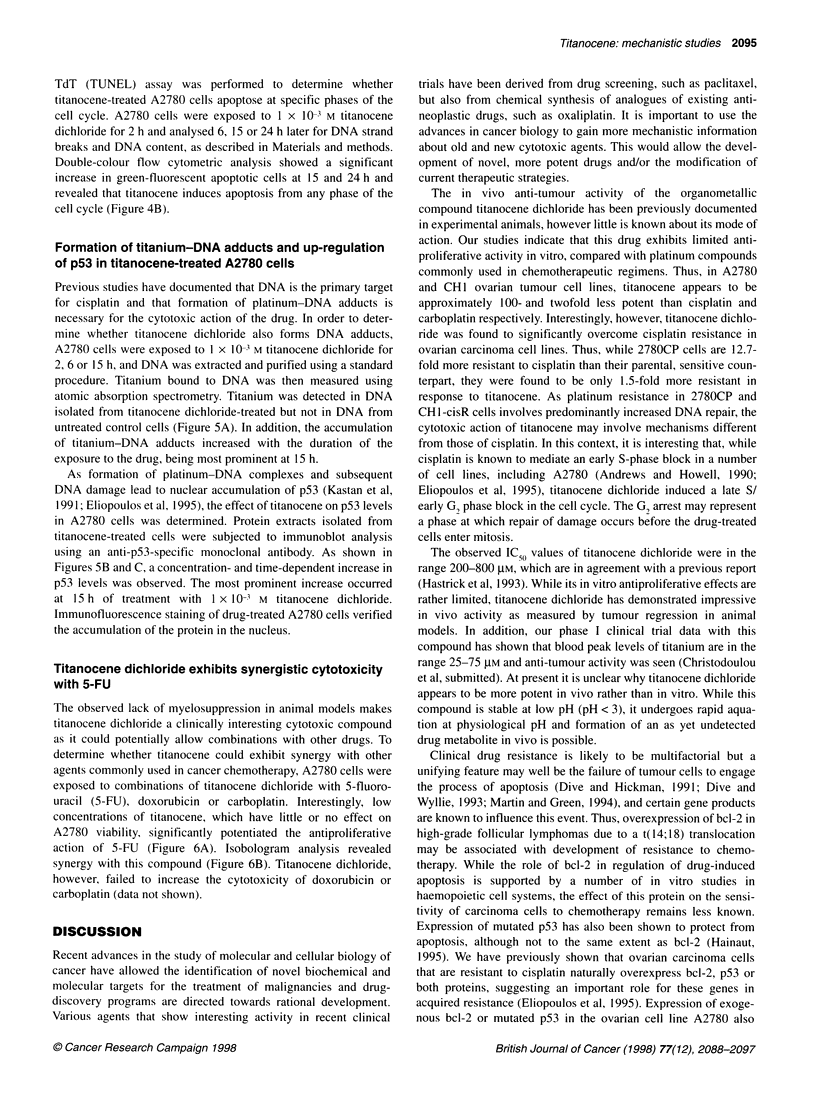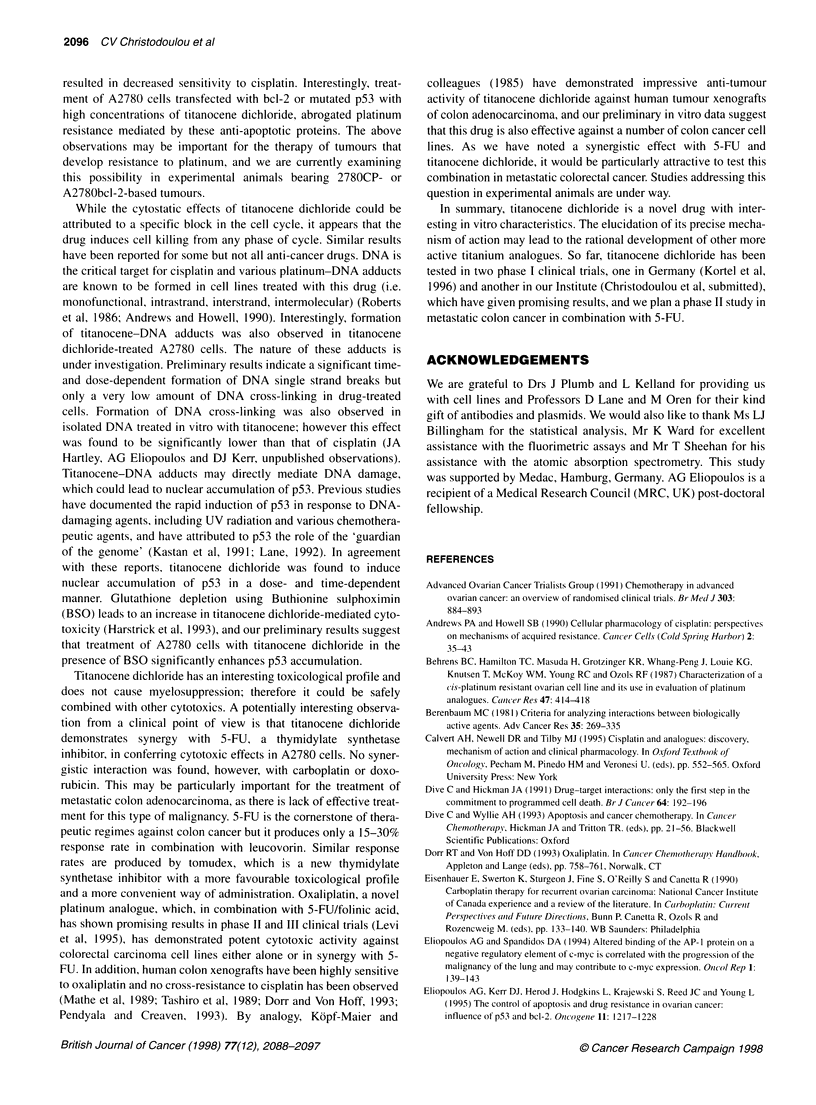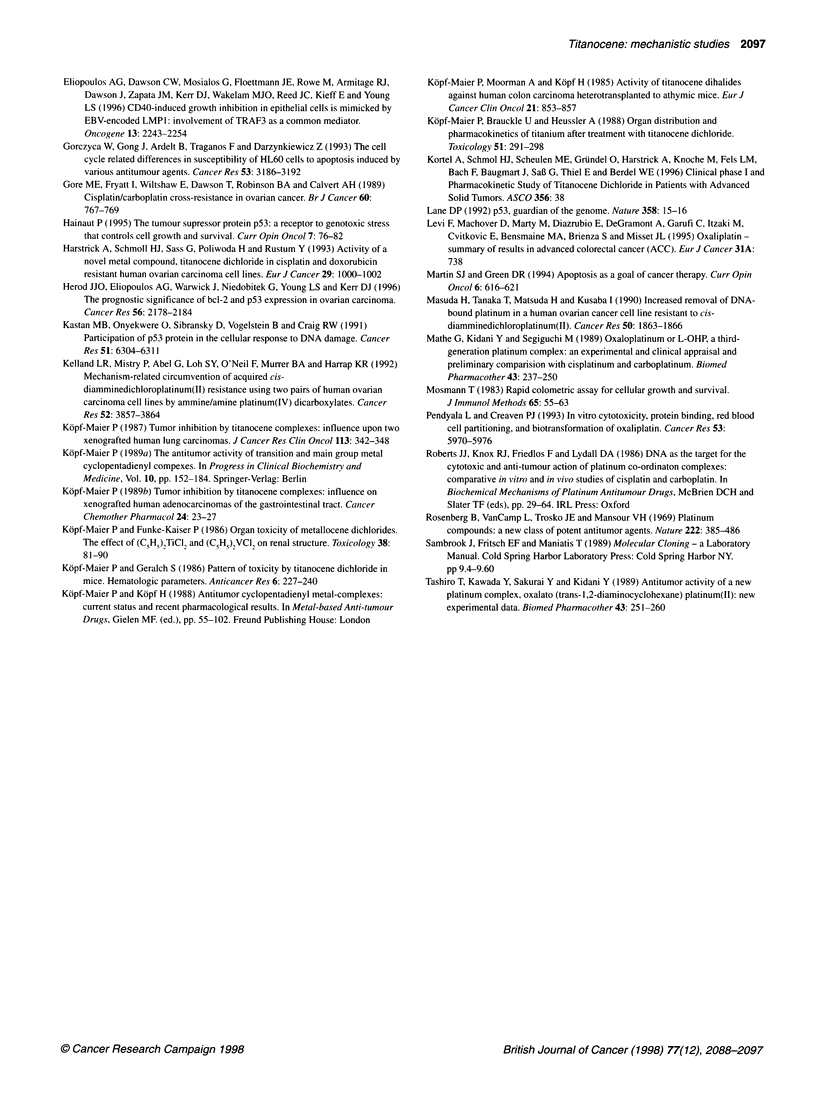# Anti-proliferative activity and mechanism of action of titanocene dichloride.

**DOI:** 10.1038/bjc.1998.352

**Published:** 1998-06

**Authors:** C. V. Christodoulou, A. G. Eliopoulos, L. S. Young, L. Hodgkins, D. R. Ferry, D. J. Kerr

**Affiliations:** CRC Institute for Cancer Studies, The University of Birmingham Medical School, UK.

## Abstract

**Images:**


British Journal of Cancer (1998) 77(12), 2088–2097
© 1998 Cancer Research Campaign

### Anti-proliferative activity and mechanism of action of titanocene dichloride

**CV Christodoulou, AG Eliopoulos, LS Young, L Hodgkins, DR Ferry and DJ Kerr**

CRC Institute for Cancer Studies, The University of Birmingham Medical School, Birmingham B15 2TA, UK

**Summary** Development of resistance to cytotoxic agents is a major limitation to their clinical use. Novel compounds are synthesized with a view to develop non-cross-resistant, less toxic and more potent activity. The detection of the anti-tumour properties of the inorganic compound cisplatin stimulated a broad search for other metal-containing complexes. Titanocene dichloride was synthesized on this basis and has shown potent anti-neoplastic activity in experimental animals. We have examined the in vitro activity of titanocene dichloride in two pairs of platinum-sensitive and resistant human ovarian carcinoma cell lines, A2780/2780CP and CH1/CH1cisR, and in mutated p53- and bcl-2-transfected clones of A2780 cells. A time- and concentration-dependent anti-proliferative effect was observed in all cell lines treated with titanocene dichloride. The drug was found to significantly overcome platinum resistance in the 2780CP and the CH1 cisR cell lines and in the bcl-2 and the mutant p53 transfectants of A2780 cells. Titanocene dichloride induced a block in late S/early $G_2$ phase of the cell cycle; however apoptotic cell death occurred from any phase of cycle. Titanium–DNA adducts were detected in A2780 cells treated with titanocene dichloride using atomic absorption spectrometry, suggesting that DNA may be a target for this drug. In agreement with this finding, p53 accumulated rapidly in drug-treated A2780 cells, indicative of a role for titanocene dichloride as a DNA-damaging agent. We have also performed studies to determine whether titanocene dichloride could demonstrate synergy with other cytotoxic agents in vitro. Isobologram analysis of cytotoxicity data obtained suggests that the combination of titanocene dichloride and 5-fluorouracil (5-FU) is synergistic. The potent in vivo anti-tumour activity of this compound, supported by the encouraging results from two phase I clinical trials, suggests that titanocene dichloride could be a promising novel chemotherapeutic agent.

**Keywords:** titanocene dichloride; organometallic compound; ovarian carcinoma cells; platinum resistance

After the discovery of its anti-tumour activity, *cis*-diammine-dichloroplatinum(II) (cisplatin, cDDP) has become one of the most widely used drugs for the treatment of cancer. Its importance was derived largely from its ability to confer complete responses in patients with advanced testicular cancer, and it was subsequently found to exhibit significant therapeutic efficacy in ovarian, uterus, bladder, and head and neck cancer (Rosenberg et al, 1969; Calvert et al, 1995). The remarkable anti-tumour effects of cisplatin coincide, however, with marked toxic effects, including neurotoxicity, nephrotoxicity and severe emetic toxicity. In addition, there is a propensity for tumours to develop resistance to platinum, which constitutes a major problem in its clinical use. The effectiveness of this drug against a wide range of human tumours, as well as its limitations, has generated a broad interest in developing new platinum complexes with lower toxicity and improved activity and spectrum.

Despite some 25 years of effort, there has been little progress to date in developing new platinum complexes with the above characteristics. Thus far, the most effective second-generation platinum compound to have been developed is carboplatin. Its spectrum of anti-tumour activity is not different from that of cisplatin, but it presents significantly reduced nephrotoxicity. However, the results of both randomized cisplatin vs carboplatin and cross-over studies performed in ovarian cancer show that both compounds are effective against essentially the same population of tumours (Gore et al, 1989; Eisenhauer et al, 1990; advanced Ovarian Cancer Trialists Group, 1991). These reports, together with accumulating in vitro data suggest that carboplatin is not capable of circumventing cisplatin resistance.

In addition to platinum, other metal-containing complexes have been synthesized and subjected to tumour screening. The anti-tumour activity of the organometallic compound titanocene dichloride [cyclopentadienyl-dichloro titanium(IV)] has been recognized and well documented in experimental animals. Thus, in a number of transplanted tumours of murine origin, such as Ehrlich ascites, sarcoma, colon adenocarcinoma, melanoma and lung carcinoma models, titanocene dichloride was highly effective (Köpf-Maier and Köpf, 1988; Köpf-Maier, 1989*a*). Moreover, the growth of xenotransplanted human tumours of colon, lung, breast and head and neck, as well as autochthonous chemically induced colorectal tumours in rats, were markedly reduced by this compound (Köpf-Maier et al, 1985; Köpf-Maier, 1987, 1989*b*). The pattern of organ toxicity of titanocene dichloride in these animals was found to be different from that of cisplatin, with hepatic toxicity being the most prominent side-effect of titanocene; unlike cisplatin and carboplatin, however, no severe nephrotoxicity or myelotoxicity was observed (Köpf-Maier and Funke-Kaiser, 1986; Köpf-Maier and Geralch, 1986; Köpf-Maier and Köpf, 1988; Köpf-Maier et al, 1988; Köpf-Maier, 1989*a*).

Titanocene dichloride has recently entered phase I clinical trial with promising results (Christodoulou et al, submitted). The dose-limiting toxicities seen following a weekly schedule of 1-h infusion were reversible nephrotoxicity and hepatotoxicity. The pharmacokinetics of titanocene dichloride appear to be complex

*Received 7 October 1997*
*Revised 9 January 1998*
*Accepted 15 January 1998*

*Correspondence to:* DJ Kerr and are described by a three-compartment model for total titanium. Peak titanium levels are in the range 25–75 μM at the maximum-tolerated dose of 140 mg $m^{-2}$ per week.

While the anti-tumour effects of titanocene dichloride on experimental animals have been well documented and the phase I clinical trial so far suggests a potential role for this compound as an anti-tumour agent, little is known about its in vitro properties and mode of action (Harstrick et al, 1993). In this study, we have demonstrated that titanocene dichloride confers potent anti-proliferative properties in vitro and may partly reverse platinum resistance in ovarian tumour cell lines. The cytostatic and cytotoxic effects of the drug could result from its observed ability to form DNA adducts and to induce cell cycle arrest and apoptosis. Understanding the mechanism of action of this novel chemotherapeutic agent may contribute to the development of more effective anti-cancer drugs.

### MATERIALS AND METHODS

### Cell lines, plasmids, transfections

The human ovarian carcinoma cell line A2780 and the acquired platinum-resistant 2780CP (Behrens et al, 1987) were kindly provided by Dr J Plumb (University of Glasgow, Glasgow, UK). These lines were continuously maintained in RPMI medium supplemented with 10% fetal calf sesum (FCS) (Flow, UK), 1% glutamine and 1% penicillin/streptomycin. The CH1 and CH1-cisR cell lines (Kelland et al, 1992) were kindly provided by Dr L Kelland (Institute for Cancer Research, Surrey, UK) and cultured in similar conditions. Development and characterization of A2780 clones expressing bcl-2 or temperature-sensitive p53 have been previously described (Eliopoulos et al, 1995).

### Anti-cancer agents and chemicals

Titanocene dichloride [cyclopentadienyl-dichloro-titanium(IV)] was provided by Medac (Hamburg, Germany). The formulation was supplied as a freeze-dried powder in vials containing 50 mg of drug. It was stored at 4°C, protected from light. The reconstitution of the compound was carried out in 0.9% sodium chloride at a concentration of 2 mg $ml^{-1}$. The final solution was strictly protected from light and was used immediately. The structure of the drug is given in Figure 1.

### MTT assay for cytotoxicity

Cytotoxicity was estimated using the colorimetric MTT conversion assay of Mosmann (1983). Briefly, cells were trypsinized and plated out at a density of 3000–5000 cells per well into a 96-well plate and allowed to attach overnight. The next day, the cells were treated for 2 h with various concentrations of titanocene dichloride and/or other cytotoxic agents, washed with phosphate-buffered saline (PBS), and 200 μl of fresh complete medium were added. Forty-eight hours later, 20 μl of 5 mg $ml^{-1}$ MTT (3,(4,4-dimethylthiazol-2-yl)-2,5-diphenyl tetrazolium bromide) (Sigma, Poole, UK) in PBS were added in each well incubated for 4 h at 37°C, and the formazan crystals fromed were dissolved in dimethyl sulphoxide (DMSO). The optical density (OD) was recorded at 550 nm on a Becton Dickinson Multisan. The $IC_{50}$ (inhibitory concentration 50%) values were the drug concentrations inducing 50% reduction in the optical density.

### [$^3$H]Thymidine incorporation assay

Cells were plated and treated as described above and pulsed for the last 4 h with 0.33 μCi [$^3$H]thymidine (Amersham). The cells were then washed twice with PBS, trypsinized and harvested using a Skatron cell harvester. [$^3$H]Thymidine incorporation was measured on a Pharmacia Betaplate counter.

### Calculation of synergy

A2780 cells were treated with various concentrations of titanocene dichloride (0, 10, 50, 100, 200 μM) in the presence of increasing concentrations of 5-fluorouracil (5-FU) (0, 10, 50, 100, 500 μM), doxorubicin (0, 0.1, 1, 5, 10 μM) or carboplatin (0, 10, 50, 100, 500 μM). Cytotoxicity was estimated using MTT conversion assays as described above. Synergy was calculated by isobologram analysis according to the method of Berenbaum (1981).

### Flow cytometric analysis of DNA content

Cells in monolayer culture were harvested, washed, fixed in 1% neutral-buffered formalin (NBF) and stained with 5 μg $ml^{-1}$ propidium iodide (PI). Fluorescence intensities were determined by quantitative flow cytometry and profiles were generated on a Coulter Epics-Profile II analyser. Coulter DNA–Cytologic Analysis software was used to calculate the cell cycle and S-phase fraction.

### Assays for the detection of apoptotic cell death

Quantification of DNA fragmentation was determined by flow cytometry using the TUNEL (TdT) assay, as previously described (Gorczyca et al, 1993; Eliopoulos et al, 1996). A2780 cells treated with titanocene dichloride and untreated controls were fixed in 2% paraformaldehyde, permeabilized in 70% ethanol and incubated in 1 × TdT buffer containing 10 U of TdT (terminal deoxynucleotidyl transferase) (Gibco, UK) and 2 mM digoxigenin-11-dUTP (Boehringer, UK) for 35 min at 37°C, followed by a 30-min incubation with 0.1% Triton X-100 and 5% non-fat milk. The cells were finally stained with 5 mg $ml^{-1}$ propidium iodide (PI), and the Pl (red, *x*-axis) and FITC (green, *y*-axis) fluorescence were then analysed on a Becton Dickinson FACScan. Specific DNA fragmentation is the percentage green fluorescence in cells treated with titanocene dichloride after subtraction of background green fluorescence in control cultures.

For the electrophoretic characterization of DNA fragmentation, approximately $10^6$ cells were lysed in a solution containing 10 mM EDTA, 50 mM Tris (pH 8), 0.5% *N*-Lauroyl-sarkosine (Sigma, Poole, UK) and 0.5 mg $ml^{-1}$ Proteinase-K (Boehringer) and incubated for 1 h in 50°C. RNAase at a concentration of 0.5 mg $ml^{-1}$ (Sigma, Poole, UK) was then added and the mixture was further incubated for 1 h. After the addition of 10 μl of a solution containing 0.01% bromophenol blue, 10 mM EDTA and 0.1% LMP agarose, the samples were analysed on a 2% agarose gel and visualized by ethidium bromide staining. Both attached and floating cells from titanocene dichloride-treated cultures were analysed.

### DNA extraction

DNA extracts from tissue specimens were prepared as described by Sambrook et al (1989), slightly modified (Eliopoulos and Spandidos, 1994). Briefly, treated cells or control cultures were

© Cancer Research Campaign 1998

**Table 1** $IC_{50}$ values for ovarian tumour cell lines treated with titanocene dichloride (TD) or cisplatin for 2 h, as determined by MTT cytotxicity assays

| Cell line | Titanocene dichloride | | Cisplatin | |
|---|---|---|---|---|
| | $IC_{50}$ (M) | Resistance factor | $IC_{50}$ (M) | Resistance factor |
| A2780 | $5.7 \times 10^{-4}$ (±1.3) | – | $4.0 \times 10^{-6}$ (±0.2) | – |
| 2780 CP | $8.4 \times 10^{-4}$ (±0.9) | 1.47 | $5.1 \times 10^{-5}$ (±0.3) | 12.7 |
| A2780bcl-2/Cl.14 | $6.4 \times 10^{-4}$ (±1.0) | 1.12 | $1.2 \times 10^{-5}$ (±0.3) | 3.0 |
| A2780bcl-2/Cl.10 | $6.6 \times 10^{-4}$ (±1.0) | 1.15 | $1.4 \times 10^{-5}$ (±0.3) | 3.5 |
| A2780tsp53/Cl.1 (37.5°C) | $6.3 \times 10^{-4}$ (±1.3) | 1.10 | $7.5 \times 10^{-6}$ (±0.5) | 1.8 |
| CH1 | $1.9 \times 10^{-4}$ (±0.9) | – | ND | – |
| CH1 cisR | $3.1 \times 10^{-4}$ (±1.1) | 1.60 | ND | – |

Values represent means of at least three independent experiments. ND, not determined.

**Figure 1** Chemical structure of titanocene dichloride [cyclopentadienyl-dichloro-titanium (IV)]

incubated overnight in lysis solution (10 mM Tris, pH 7.8, 150 mM sodium chloride, 0.5% sodium dodecyl sulphate (SDS) and 100 mg ml$^{-1}$ proteinase-K) at 37°C. After two phenol–chloroform extractions, the aqueous phase was removed and incubated for 2 h with 100 μg ml$^{-1}$ pancreatic RNAase (Sigma) at 37°C and for an additional 2-h period with 100 μg ml$^{-1}$ proteinase-K (Boehringer) followed by phenol–chloroform extraction twice. The aqueous phase was then removed and the DNA was precipitated with ethanol. The DNA was washed with 70% ethanol, dried briefly, resuspended in sterile water and estimated photometrically at 260 nm using an LKB 4054 spectrophotometer.

### Detection of titanium–DNA adducts

DNA-bound titanium was measured using electrothermal atomization atomic absorption spectrometry on a Varian Spectra AA-400 equipped with deuterium background correction. Analysis was performed directly after dilution of the DNA solutions with 0.1% $HNO_3$. For calibration identically-treated matrix-matched standards were used. Standards were prepared using inorganic titanium (BDH, Poole, UK), the analytical behaviour of this form of the element having been found to match that of titanium present in solutions of titanocene dichloride in 0.1% nitric acid.

### Protein extraction and Western blot analysis

Cell protein extracts were prepared by lysing cells into a buffer containing 50 mM Tris (pH 6.8), 0.15 M β-mercaptoethanol and 9 M urea and subsequent sonication. Protein concentration was estimated using the Biorad protein assay. Then, 100 μg of total protein was separated on a 10% SDS–polyacrylamide gel in a Tris-glycine running buffer. After electrophoresis, the proteins were electroblotted onto nitrocellulose (85 V, 5 h). The blots were blocked in 5% non-fat milk in PBS-Tween (blotto) and incubated overnight with antibody properly diluted in blocking solution. Immune complexes were detected with [$^{125}$I]Protein-A (Amersham, UK) after incubation with appropriate immunoglobulin (IgG). Antibody used was the PAb1801 mouse anti-human p53 (gift from Professor D Lane, Department of Biochemistry, University of Dundee, UK).

### RESULTS

### Titanocene dichloride exhibits anti-proliferative activity in vitro

In order to determine whether titanocene dichloride confers anti-proliferative properties in vitro, A2780 ovarian carcinoma cells were treated for 2 h with various drug concentrations ($1 \times 10^{-6}$–$2 \times 10^{-3}$ M), and cell viability was assessed 48 h later by MTT conversion assays. Analysis of the dose–response curves (Figure 2) indicated that titanocene dichloride is effective in a rather narrow range of concentrations, as determined by the inability of the drug to induce cell killing in concentrations lower than $1 \times 10^{-4}$ M and by the steep pattern of the slope of the cytotoxicity curve. Under these experimental conditions, the $IC_{50}$ value for the titanocene-treated A2780 cells was 5.7 (±1.3) × $10^{-4}$ M (Table 1), while the corresponding values for cisplatin or carboplatin-treated A2780 cells were 4.0 (±0.2) × $10^{-6}$ M and 2.5 (±0.6) × $10^{-4}$ M respectively (Eliopoulos et al, 1995 and data not shown). The $IC_{50}$ values after a 48-h continuous exposure to titanocene dichloride, cisplatin or carboplatin were also determined and were 2.8 (±1.0) × $10^{-4}$, 0.8 (±0.4) × $10^{-6}$ and 1.2 (±0.5) × $10^{-4}$ M respectively. Similar results were obtained when [$^{3}$H]thymidine incorporation assays were performed (Figure 2C). In addition, treatment of CH1 ovarian tumour cells with titanocene dichloride induced a concentration-dependent cytotoxic effect, with an $IC_{50}$ value of 1.9 × $10^{-4}$ M (Table 1). The pattern of dose–response curve for drug-treated CH1 cells was similar to that of A2780 (data not shown). The above results suggest that titanocene dichloride confers antiproliferative properties in vitro, however the drug concentrations necessary to inhibit cell growth are significantly higher than those of cisplatin.

© Cancer Research Campaign 1998

**Figure 2** (**A** and **B**) Cytotoxicity curves from typical MTT assays showing the effect of titanocene dichloride (TD) on the viability of A2780 ovarian cell line, a bcl-2 transfectant (A2780bcl-2/Cl.14), a tsp53 transfectant (A2780tsp53/Cl.1) expressing mutated p53 and the resistant variant 2780CP. (**C**) Growth curves from a typical [$^3$H]thymidine incorporation assay showing the effect of TD on the proliferation of A2780, 2780CP and A2780bcl-2/Cl.14 cells. The shift of the curve to the right is characteristic of an increase in viability. At least three independent experiments were performed and gave similar results

### Titanocene dichloride significantly overcomes platinum resistance in vitro

In order to determine whether titanocene dichloride overcomes platinum resistance in vitro, the anti-proliferative effects of the drug on cisplatin-resistant A2780 and CH1 variants (2780CP and CH1 cisR respectively) were examined. As ovarian tumours often develop resistance to platinum, these lines present a good model for screening novel compounds with a potential of overcoming resistance. In addition, the above cell lines have been well characterized in terms of mechanism of resistance. Thus, cisplatin resistance in 2780CP and CH1cisR cells appears to be associated with increased DNA repair (Masuda et al, 1990; Kelland et al, 1992), although we have also noticed increased glutathione levels in 2780CP cells (AG Eliopoulos, unpublished observations).

When 2780CP cells were treated with titanocene dichloride, a concentration-dependent antiproliferative effect was observed using MTT conversion or [$^3$H]thymidine incorporation assays (Figures 2A and C). The $IC_{50}$ value, as determined by MTT assays was 8.4 ($\pm$0.9)$\times10^{-4}$ M (Table 1), while the corresponding value for cisplatin-treated 2780CP cells has been previously found to be 5.1 ($\pm$0.3) $\times$ $10^{-5}$ M (Eliopoulos et al, 1995). Thus, while 2780CP cells are 12.7 times more resistant to platinum than their sensitive parental counterpart, the cisplatin-resistant variant appears to be only 1.5-fold more resistant to titanocene dichloride compared with A2780 cells. Similar results were obtained for CH1/CH1cisR cells and are summarized in Table 1. The observed dramatic decrease in resistance factor suggests that titanocene dichloride confers a potent antiproliferative effect on platinum-resistant ovarian tumour cell lines.

Over the last few years, there has been increasing evidence that expression of certain anti-apoptotic genes, such as *bcl-2* and *p53*, may affect the cellular response to chemotherapy and therefore modulate the sensitivity of cells to anti-cancer drugs (Dive and Hickman, 1991; Dive and Wyllie, 1993; Martin and Green, 1994). Thus, we have recently shown that bcl-2 and p53 are frequently expressed in ovarian carcinomas (Herod et al, 1996), and cisplatin-resistant ovarian tumour cell lines have elevated levels of these anti-apoptotic proteins compared with their normal counterparts (Eliopoulos et al, 1995). In addition, transfection of A2780 cells with a bcl-2- or a temperature-sensitive mutant p53 (ts p53)-expressing plasmid increased platinum resistance by 2.1 to 3.5- and 1.7- to 2.4-fold respectively (Eliopoulos et al, 1995). Examination of two of these bcl-2 clones (A2780bcl-2/Cl.10 and Cl. 14) for response to titanocene dichloride showed that this drug may be very effective against resistance conferred by bcl-2. In each experiment performed, the dose–response curve of titanocene-treated A2780bcl-2/Cl. 14 vs A2780 cells was consistently shifted to the right (Figure 2B and C), which suggests that bcl-2 confers some degree of resistance; however the difference in $IC_{50}$ values is too small to support a biological significance. Thus, while cisplatin-treated A2780bcl-2 cells were approximately three-fold more resistant than the parental line, the resistant factor for titanocene-treated bcl-2-expressing cells was 1.2 (Table 1), which suggests that titanocene dichloride may overcome bcl-2-mediated platinum resistance.

As mutated p53 confers a survival advantage against chemotherapy (Hainaut, 1995), the response of tsp53-transfected A2780 cells (A2780tsp53/Cl.1) to titanocene was examined using MTT assays. At the permissive temperature of 37.5°C, at which the p53 protein is predominantly in the mutant conformation, the

© Cancer Research Campaign 1998

**Figure 3** (**A**) DNA content of A2780 cells treated with $1 \times 10^{-3}$ M titanocene dichloride (TD) for 2 h and collected at various time points post treatment (6, 15, 24, 30, 48 h), as assessed by flow cytometry. TD induced a significant block in late S-/early $G_2$ phase. (**B**) Cell cycle changes in A2780, 2780CP and A2780bcl-2/Cl.14 after a 2-h exposure to $1 \times 10^{-3}$ M TD. The percentage of cells in $G_1$, S and $G_2$/M phases of the cell cycle is shown. Five independent experiments were performed and gave similar results

© Cancer Research Campaign 1998

**Figure 4** (**A**) Formation of low-molecular-weight DNA in A2780 ovarian tumour cells at 24 h post treatment with $1 \times 10^{-5}$, $1 \times 10^{-4}$ or $1 \times 10^{-3}$ M titanocene dichloride (TD) for 2 h. DNA ladders were not detected in control untreated cells (lane 1). (**B**) Apoptotic A2780 cells at 6, 15 and 24 h after a 2 h exposure to $1 \times 10^{-3}$ M TD, as determined with the TdT (TUNEL) assay. The A2780 cells apoptose from any phase of the cell cycle in response to titanocene treatment. The percentage of apoptotic cells is given on the top right-hand side of the graphs dose–response curves for mutated p53-expressing vs control A2780 cells were almost identical (Figure 2B). As we have previously shown that A2780tsp53/Cl.1 cells confer a 1.8-fold increase in cisplatin resistance (Eliopoulos et al, 1995), these results suggest that titanocene dichloride overcomes mutated p53-mediated platinum resistance in ovarian tumour cell lines.

*© Cancer Research Campaign 1998* 

**Figure 5** (**A**) Kinetics of formation of titanium–DNA adducts. A2780 cells were treated with 1 × 10$^{-3}$ M titanocene dichloride (TD) for 0, 2, 6 or 15 h and isolated DNA was subjected to atomic absorption spectrometry for the detection of titanium bound to DNA. The data are representative of two independent experiments. (**B** and **C**) Induction of p53 protein levels in response to titanocene dichloride treatment. A2780 cells were collected at 12 h post treatment with various drug concentrations and analysed for p53 expression by immunoblot (**B**). Alternatively, A2780 cells were exposed to 1 × 10$^{-3}$ M TD for 2 h and analysed at 2, 6, 12 or 24 h later for p53 levels (**C**). Data are representative of at least three independent experiments

**Figure 6** (**A**) Cytotoxicity curves from a representative MTT assay showing the effects of increasing concentrations of 5-fluorouracil (5-FU) in combination with various concentrations of titanocene dichloride (TD) on the viability of A2780 ovarian cell line. In this experiment, the IC$_{50}$ values for the two drugs were 4.9 and 5.1×10$^{-4}$ M respectively. (**B**) TD and 5-FU interaction data presented by isobol plot. After determining the IC$_{50}$ concentrations for the individual drugs, a range of fractions of the IC$_{50}$ value for TD was selected. For each of these fractions, we then determined the amount of 5-FU necessary to restore the total drug effect to 50% growth inhibition. The isobol plots the amounts of 5-FU (as a fraction of its IC$_{50}$) that give the 50% inhibition of cell growth. The IC$_{50}$ values reported represent the mean values of four independent experiments using various combinations of TD and 5-FU for the treatment of human ovarian carcinoma cells A2780. The 5-FU and the TD concentrations used in these experiments varied from 1 × 10$^{-5}$ to 1 × 10$^{-3}$ M

IC$_{50}$ values (Table 1) were calculated and subjected to two-way analysis of variance. This was carried out to assess the relative resistance of the different cell lines to titanocene dichloride. This analysis allows the variation between the cell lines that are under investigation while adjusting for the variation between the experiments. Multiple comparison tests showed that the 2780CP line has significantly higher IC$_{50}$ value compared with the other three lines (A2780, A2780bcl-2 and A2780tsp53), which do not differ. The above difference was observed after 2 h ($P = 0.01$ for MTT assays, $P = 0.08$ for [$^3$H]thymidine) and 48 h drug exposure ($P = 0.002$).

### Titanocene dichloride induces cell cycle arrest in ovarian tumour cell lines

In order to determine whether the anti-proliferative effects of titanocene dichloride involve growth arrest at specific phases of the cell cycle, A2780 cells were collected at various time points (0, 6, 15, 24, 30, 48 h) after treatment with 1x10$^{-3}$ M titanocene and analysed for their DNA content using flow cytometry. As shown in Figure 3, titanocene dichloride induced a significant block in late S/early $G_2$ phase of the cell cycle at 15 and 24 h after treatment. This block was however transient and cells returned to normal cycling by 48 h after exposure to the drug. Thus, the percentage of A2780 cells in $G_2$/M phase increased from 10.7 at 0 h to 14.7 and 33.6 at 15 and 24 h respectively. 2780CP and A2780bcl-2/Cl.14 cells were also examined for cell cycle block upon titanocene treatment and gave similar results (Figure 3B).

### Titanocene dichloride induces apoptosis in ovarian tumour cell lines

Apoptosis is a mode of cell death observed in a variety of cell types after treatment with chemotherapeutic agents. In order to determine whether titanocene dichloride also induces apoptosis in vitro, A2780 cells were treated for 2 h with 1 × 10$^{-5}$ M, 1 × 10$^{-4}$ M or 1 × 10$^{-3}$ M titanocene and analysed 24 h later for DNA fragmentation using agarose gel electrophoresis. Formation of small-molecular-weight DNA (DNA ladders) is a hallmark of apoptotic cell death. As shown in Figure 4A, titanocene dichloride induced formation of DNA ladders at 1 × 10$^{-3}$ M and to a lesser extent at 1 × 10$^{-4}$ M but not at the lower concentration of 1 × 10$^{-5}$ M. DNA ladders were not detected in control, untreated cultures (Figure 4A, lane 1).

As this agarose electrophoresis assay does not allow correlation between DNA fragmentation and position in the cell cycle, the

© Cancer Research Campaign 1998

TdT (TUNEL) assay was performed to determine whether titanocene-treated A2780 cells apoptose at specific phases of the cell cycle. A2780 cells were exposed to $1 \times 10^{-3}$ M titanocene dichloride for 2 h and analysed 6, 15 or 24 h later for DNA strand breaks and DNA content, as described in Materials and methods. Double-colour flow cytometric analysis showed a significant increase in green-fluorescent apoptotic cells at 15 and 24 h and revealed that titanocene induces apoptosis from any phase of the cell cycle (Figure 4B).

### Formation of titanium–DNA adducts and up-regulation of p53 in titanocene-treated A2780 cells

Previous studies have documented that DNA is the primary target for cisplatin and that formation of platinum–DNA adducts is necessary for the cytotoxic action of the drug. In order to determine whether titanocene dichloride also forms DNA adducts, A2780 cells were exposed to $1 \times 10^{-3}$ M titanocene dichloride for 2, 6 or 15 h, and DNA was extracted and purified using a standard procedure. Titanium bound to DNA was then measured using atomic absorption spectrometry. Titanium was detected in DNA isolated from titanocene dichloride-treated but not in DNA from untreated control cells (Figure 5A). In addition, the accumulation of titanium–DNA adducts increased with the duration of the exposure to the drug, being most prominent at 15 h.

As formation of platinum–DNA complexes and subsequent DNA damage lead to nuclear accumulation of p53 (Kastan et al, 1991; Eliopoulos et al, 1995), the effect of titanocene on p53 levels in A2780 cells was determined. Protein extracts isolated from titanocene-treated cells were subjected to immunoblot analysis using an anti-p53-specific monoclonal antibody. As shown in Figures 5B and C, a concentration- and time-dependent increase in p53 levels was observed. The most prominent increase occurred at 15 h of treatment with $1 \times 10^{-3}$ M titanocene dichloride. Immunofluorescence staining of drug-treated A2780 cells verified the accumulation of the protein in the nucleus.

### Titanocene dichloride exhibits synergistic cytotoxicity with 5-FU

The observed lack of myelosuppression in animal models makes titanocene dichloride a clinically interesting cytotoxic compound as it could potentially allow combinations with other drugs. To determine whether titanocene could exhibit synergy with other agents commonly used in cancer chemotherapy, A2780 cells were exposed to combinations of titanocene dichloride with 5-fluorouracil (5-FU), doxorubicin or carboplatin. Interestingly, low concentrations of titanocene, which have little or no effect on A2780 viability, significantly potentiated the antiproliferative action of 5-FU (Figure 6A). Isobologram analysis revealed synergy with this compound (Figure 6B). Titanocene dichloride, however, failed to increase the cytotoxicity of doxorubicin or carboplatin (data not shown).

### DISCUSSION

Recent advances in the study of molecular and cellular biology of cancer have allowed the identification of novel biochemical and molecular targets for the treatment of malignancies and drug-discovery programs are directed towards rational development. Various agents that show interesting activity in recent clinical trials have been derived from drug screening, such as paclitaxel, but also from chemical synthesis of analogues of existing anti-neoplastic drugs, such as oxaliplatin. It is important to use the advances in cancer biology to gain more mechanistic information about old and new cytotoxic agents. This would allow the development of novel, more potent drugs and/or the modification of current therapeutic strategies.

The in vivo anti-tumour activity of the organometallic compound titanocene dichloride has been previously documented in experimental animals, however little is known about its mode of action. Our studies indicate that this drug exhibits limited anti-proliferative activity in vitro, compared with platinum compounds commonly used in chemotherapeutic regimens. Thus, in A2780 and CH1 ovarian tumour cell lines, titanocene appears to be approximately 100- and twofold less potent than cisplatin and carboplatin respectively. Interestingly, however, titanocene dichloride was found to significantly overcome cisplatin resistance in ovarian carcinoma cell lines. Thus, while 2780CP cells are 12.7-fold more resistant to cisplatin than their parental, sensitive counterpart, they were found to be only 1.5-fold more resistant in response to titanocene. As platinum resistance in 2780CP and CH1-cisR cells involves predominantly increased DNA repair, the cytotoxic action of titanocene may involve mechanisms different from those of cisplatin. In this context, it is interesting that, while cisplatin is known to mediate an early S-phase block in a number of cell lines, including A2780 (Andrews and Howell, 1990; Eliopoulos et al, 1995), titanocene dichloride induced a late S/early $G_2$ phase block in the cell cycle. The $G_2$ arrest may represent a phase at which repair of damage occurs before the drug-treated cells enter mitosis.

The observed $IC_{50}$ values of titanocene dichloride were in the range 200–800 μM, which are in agreement with a previous report (Hastrick et al, 1993). While its in vitro antiproliferative effects are rather limited, titanocene dichloride has demonstrated impressive in vivo activity as measured by tumour regression in animal models. In addition, our phase I clinical trial data with this compound has shown that blood peak levels of titanium are in the range 25–75 μM and anti-tumour activity was seen (Christodoulou et al, submitted). At present it is unclear why titanocene dichloride appears to be more potent in vivo rather than in vitro. While this compound is stable at low pH ($pH < 3$), it undergoes rapid aquation at physiological pH and formation of an as yet undetected drug metabolite in vivo is possible.

Clinical drug resistance is likely to be multifactorial but a unifying feature may well be the failure of tumour cells to engage the process of apoptosis (Dive and Hickman, 1991; Dive and Wyllie, 1993; Martin and Green, 1994), and certain gene products are known to influence this event. Thus, overexpression of bcl-2 in high-grade follicular lymphomas due to a t(14;18) translocation may be associated with development of resistance to chemotherapy. While the role of bcl-2 in regulation of drug-induced apoptosis is supported by a number of in vitro studies in haemopoietic cell systems, the effect of this protein on the sensitivity of carcinoma cells to chemotherapy remains less known. Expression of mutated p53 has also been shown to protect from apoptosis, although not to the same extent as bcl-2 (Hainaut, 1995). We have previously shown that ovarian carcinoma cells that are resistant to cisplatin naturally overexpress bcl-2, p53 or both proteins, suggesting an important role for these genes in acquired resistance (Eliopoulos et al, 1995). Expression of exogenous bcl-2 or mutated p53 in the ovarian cell line A2780 also

© Cancer Research Campaign 1998 

resulted in decreased sensitivity to cisplatin. Interestingly, treatment of A2780 cells transfected with bcl-2 or mutated p53 with high concentrations of titanocene dichloride, abrogated platinum resistance mediated by these anti-apoptotic proteins. The above observations may be important for the therapy of tumours that develop resistance to platinum, and we are currently examining this possibility in experimental animals bearing 2780CP- or A2780bcl-2-based tumours.

While the cytostatic effects of titanocene dichloride could be attributed to a specific block in the cell cycle, it appears that the drug induces cell killing from any phase of cycle. Similar results have been reported for some but not all anti-cancer drugs. DNA is the critical target for cisplatin and various platinum–DNA adducts are known to be formed in cell lines treated with this drug (i.e. monofunctional, intrastrand, interstrand, intermolecular) (Roberts et al, 1986; Andrews and Howell, 1990). Interestingly, formation of titanocene–DNA adducts was also observed in titanocene dichloride-treated A2780 cells. The nature of these adducts is under investigation. Preliminary results indicate a significant time- and dose-dependent formation of DNA single strand breaks but only a very low amount of DNA cross-linking in drug-treated cells. Formation of DNA cross-linking was also observed in isolated DNA treated in vitro with titanocene; however this effect was found to be significantly lower than that of cisplatin (JA Hartley, AG Eliopoulos and DJ Kerr, unpublished observations). Titanocene–DNA adducts may directly mediate DNA damage, which could lead to nuclear accumulation of p53. Previous studies have documented the rapid induction of p53 in response to DNA-damaging agents, including UV radiation and various chemotherapeutic agents, and have attributed to p53 the role of the 'guardian of the genome' (Kastan et al, 1991; Lane, 1992). In agreement with these reports, titanocene dichloride was found to induce nuclear accumulation of p53 in a dose- and time-dependent manner. Glutathione depletion using Buthionine sulphoximin (BSO) leads to an increase in titanocene dichloride-mediated cytotoxicity (Harstrick et al, 1993), and our preliminary results suggest that treatment of A2780 cells with titanocene dichloride in the presence of BSO significantly enhances p53 accumulation.

Titanocene dichloride has an interesting toxicological profile and does not cause myelosuppression; therefore it could be safely combined with other cytotoxics. A potentially interesting observation from a clinical point of view is that titanocene dichloride demonstrates synergy with 5-FU, a thymidylate synthetase inhibitor, in conferring cytotoxic effects in A2780 cells. No synergistic interaction was found, however, with carboplatin or doxorubicin. This may be particularly important for the treatment of metastatic colon adenocarcinoma, as there is lack of effective treatment for this type of malignancy. 5-FU is the cornerstone of therapeutic regimes against colon cancer but it produces only a 15–30% response rate in combination with leucovorin. Similar response rates are produced by tomudex, which is a new thymidylate synthetase inhibitor with a more favourable toxicological profile and a more convenient way of administration. Oxaliplatin, a novel platinum analogue, which, in combination with 5-FU/folinic acid, has shown promising results in phase II and III clinical trials (Levi et al, 1995), has demonstrated potent cytotoxic activity against colorectal carcinoma cell lines either alone or in synergy with 5-FU. In addition, human colon xenografts have been highly sensitive to oxaliplatin and no cross-resistance to cisplatin has been observed (Mathe et al, 1989; Tashiro et al, 1989; Dorr and Von Hoff, 1993; Pendyala and Creaven, 1993). By analogy, Köpf-Maier and colleagues (1985) have demonstrated impressive anti-tumour activity of titanocene dichloride against human tumour xenografts of colon adenocarcinoma, and our preliminary in vitro data suggest that this drug is also effective against a number of colon cancer cell lines. As we have noted a synergistic effect with 5-FU and titanocene dichloride, it would be particularly attractive to test this combination in metastatic colorectal cancer. Studies addressing this question in experimental animals are under way.

In summary, titanocene dichloride is a novel drug with interesting in vitro characteristics. The elucidation of its precise mechanism of action may lead to the rational development of other more active titanium analogues. So far, titanocene dichloride has been tested in two phase I clinical trials, one in Germany (Kortel et al, 1996) and another in our Institute (Christodoulou et al, submitted), which have given promising results, and we plan a phase II study in metastatic colon cancer in combination with 5-FU.

### ACKNOWLEDGEMENTS

We are grateful to Drs J Plumb and L Kelland for providing us with cell lines and Professors D Lane and M Oren for their kind gift of antibodies and plasmids. We would also like to thank Ms LJ Billingham for the statistical analysis, Mr K Ward for excellent assistance with the fluorimetric assays and Mr T Sheehan for his assistance with the atomic absorption spectrometry. This study was supported by Medac, Hamburg, Germany. AG Eliopoulos is a recipient of a Medical Research Council (MRC, UK) post-doctoral fellowship.

### REFERENCES

Advanced Ovarian Cancer Trialists Group (1991) Chemotherapy in advanced ovarian cancer: an overview of randomised clinical trials. *Br Med J* **303**: 884–893

Andrews PA and Howell SB (1990) Cellular pharmacology of cisplatin: perspectives on mechanisms of acquired resistance. *Cancer Cells (Cold Spring Harbor)* **2**: 35–43

Behrens BC, Hamilton TC, Masuda H, Grotzinger KR, Whang-Peng J, Louie KG, Knutsen T, McKoy WM, Young RC and Ozols RF (1987) Characterization of a *cis*-platinum resistant ovarian cell line and its use in evaluation of platinum analogues. *Cancer Res* **47**: 414–418

Berenbaum MC (1981) Criteria for analyzing interactions between biologically active agents. Adv Cancer Res **35**: 269–335

Calvert AH, Newell DR and Tilby MJ (1995) Cisplatin and analogues: discovery, mechanism of action and clinical pharmacology. In *Oxford Textbook of Oncology*, Pecham M, Pinedo HM and Veronesi U. (eds), pp. 552–565. Oxford University Press: New York

Dive C and Hickman JA (1991) Drug–target interactions: only the first step in the commitment to programmed cell death. *Br J Cancer* **64**: 192–196

Dive C and Wyllie AH (1993) Apoptosis and cancer chemotherapy. In *Cancer Chemotherapy*, Hickman JA and Tritton TR. (eds), pp. 21–56. Blackwell Scientific Publications: Oxford

Dorr RT and Von Hoff DD (1993) Oxaliplatin. In *Cancer Chemotherapy Handbook*, Appleton and Lange (eds), pp. 758–761, Norwalk, CT

Eisenhauer E, Swerton K, Sturgeon J, Fine S, O'Reilly S and Canetta R (1990) Carboplatin therapy for recurrent ovarian carcinoma: National Cancer Institute of Canada experience and a review of the literature. In *Carboplatin: Current Perspectives and Future Directions*, Bunn P, Canetta R, Ozols R and Rozencweig M. (eds), pp. 133–140. WB Saunders: Philadelphia

Eliopoulos AG and Spandidos DA (1994) Altered binding of the AP-1 protein on a negative regulatory element of c-myc is correlated with the progression of the malignancy of the lung and may contribute to c-myc expression. *Oncol Rep* **1**: 139–143

Eliopoulos AG, Kerr DJ, Herod J, Hodgkins L, Krajewski S, Reed JC and Young L (1995) The control of apoptosis and drug resistance in ovarian cancer: influence of p53 and bcl-2. *Oncogene* **11**: 1217–1228

© Cancer Research Campaign 1998

Eliopoulos AG, Dawson CW, Mosialos G, Floettmann JE, Rowe M, Armitage RJ, Dawson J, Zapata JM, Kerr DJ, Wakelam MJO, Reed JC, Kieff E and Young LS (1996) CD40-induced growth inhibition in epithelial cells is mimicked by EBV-encoded LMP1: involvement of TRAF3 as a common mediator. *Oncogene* **13**: 2243–2254

Gorczyca W, Gong J, Ardelt B, Traganos F and Darzynkiewicz Z (1993) The cell cycle related differences in susceptibility of HL60 cells to apoptosis induced by various antitumour agents. *Cancer Res* **53**: 3186–3192

Gore ME, Fryatt I, Wiltshaw E, Dawson T, Robinson BA and Calvert AH (1989) Cisplatin/carboplatin cross-resistance in ovarian cancer. *Br J Cancer* **60**: 767–769

Hainaut P (1995) The tumour supressor protein p53: a receptor to genotoxic stress that controls cell growth and survival. *Curr Opin Oncol* **7**: 76–82

Harstrick A, Schmoll HJ, Sass G, Poliwoda H and Rustum Y (1993) Activity of a novel metal compound, titanocene dichloride in cisplatin and doxorubicin resistant human ovarian carcinoma cell lines. *Eur J Cancer* **29**: 1000–1002

Herod JJO, Eliopoulos AG, Warwick J, Niedobitek G, Young LS and Kerr DJ (1996) The prognostic significance of bcl-2 and p53 expression in ovarian carcinoma. *Cancer Res* **56**: 2178–2184

Kastan MB, Onyekwere O, Sibransky D, Vogelstein B and Craig RW (1991) Participation of p53 protein in the cellular response to DNA damage. *Cancer Res* **51**: 6304–6311

Kelland LR, Mistry P, Abel G, Loh SY, O'Neil F, Murrer BA and Harrap KR (1992) Mechanism-related circumvention of acquired *cis*-diamminedichloroplatinum(II) resistance using two pairs of human ovarian carcinoma cell lines by ammine/amine platinum(IV) dicarboxylates. *Cancer Res* **52**: 3857–3864

Köpf-Maier P (1987) Tumor inhibition by titanocene complexes: influence upon two xenografted human lung carcinomas. *J Cancer Res Clin Oncol* **113**: 342–348

Köpf-Maier P (1989*a*) The antitumor activity of transition and main group metal cyclopentadienyl compexes. In *Progress in Clinical Biochemistry and Medicine*, Vol. **10**, pp. 152–184. Springer-Verlag: Berlin

Köpf-Maier P (1989*b*) Tumor inhibition by titanocene complexes: influence on xenografted human adenocarcinomas of the gastrointestinal tract. *Cancer Chemother Pharmacol* **24**: 23–27

Köpf-Maier P and Funke-Kaiser P (1986) Organ toxicity of metallocene dichlorides. The effect of $(C_5H_5)_2TiCl_2$ and $(C_5H_5)_2VCl_2$ on renal structure. *Toxicology* **38**: 81–90

Köpf-Maier P and Geralch S (1986) Pattern of toxicity by titanocene dichloride in mice. Hematologic parameters. *Anticancer Res* **6**: 227–240

Köpf-Maier P and Köpf H (1988) Antitumor cyclopentadienyl metal-complexes: current status and recent pharmacological results. In *Metal-based Anti-tumour Drugs*, Gielen MF. (ed.), pp. 55–102. Freund Publishing House: London

Köpf-Maier P, Moorman A and Köpf H (1985) Activity of titanocene dihalides against human colon carcinoma heterotransplanted to athymic mice. *Eur J Cancer Clin Oncol* **21**: 853–857

Köpf-Maier P, Brauckle U and Heussler A (1988) Organ distribution and pharmacokinetics of titanium after treatment with titanocene dichloride. *Toxicology* **51**: 291–298

Kortel A, Schmol HJ, Scheulen ME, Gründel O, Harstrick A, Knoche M, Fels LM, Bach F, Baugmart J, Saß G, Thiel E and Berdel WE (1996) Clinical phase I and Pharmacokinetic Study of Titanocene Dichloride in Patients with Advanced Solid Tumors. *ASCO* **356**: 38

Lane DP (1992) p53, guardian of the genome. *Nature* **358**: 15–16

Levi F, Machover D, Marty M, Diazrubio E, DeGramont A, Garufi C, Itzaki M, Cvitkovic E, Bensmaine MA, Brienza S and Misset JL (1995) Oxaliplatin – summary of results in advanced colorectal cancer (ACC). *Eur J Cancer* **31A**: 738

Martin SJ and Green DR (1994) Apoptosis as a goal of cancer therapy. *Curr Opin Oncol* **6**: 616–621

Masuda H, Tanaka T, Matsuda H and Kusaba I (1990) Increased removal of DNA-bound platinum in a human ovarian cancer cell line resistant to *cis*-diamminedichloroplatinum(II). *Cancer Res* **50**: 1863–1866

Mathe G, Kidani Y and Segiguchi M (1989) Oxaloplatinum or L-OHP, a third-generation platinum complex: an experimental and clinical appraisal and preliminary comparision with cisplatinum and carboplatinum. *Biomed Pharmacother* **43**: 237–250

Mosmann T (1983) Rapid colometric assay for cellular growth and survival. *J Immunol Methods* **65**: 55–63

Pendyala L and Creaven PJ (1993) In vitro cytotoxicity, protein binding, red blood cell partitioning, and biotransformation of oxaliplatin. *Cancer Res* **53**: 5970–5976

Roberts JJ, Knox RJ, Friedlos F and Lydall DA (1986) DNA as the target for the cytotoxic and anti-tumour action of platinum co-ordinaton complexes: comparative *in vitro* and *in vivo* studies of cisplatin and carboplatin. In *Biochemical Mechanisms of Platinum Antitumour Drugs*, McBrien DCH and Slater TF (eds), pp. 29–64. IRL Press: Oxford

Rosenberg B, VanCamp L, Trosko JE and Mansour VH (1969) Platinum compounds: a new class of potent antitumor agents. *Nature* **222**: 385–486

Sambrook J, Fritsch EF and Maniatis T (1989) *Molecular Cloning* – a Laboratory Manual. Cold Spring Harbor Laboratory Press: Cold Spring Harbor NY. pp 9.4–9.60

Tashiro T, Kawada Y, Sakurai Y and Kidani Y (1989) Antitumor activity of a new platinum complex, oxalato (trans-1,2-diaminocyclohexane) platinum(II): new experimental data. *Biomed Pharmacother* **43**: 251–260

© Cancer Research Campaign 1998